# A comparison between femoral neck and LS-BMD with LS-TBS in T2DM patients: a case control study

**DOI:** 10.1186/s12891-021-04471-7

**Published:** 2021-06-25

**Authors:** Negar Delbari, Alireza Rajaei, Mahbobeh Oroei, Arman Ahmadzadeh, Faraneh Farsad

**Affiliations:** 1 Department of Rheumatology, Loghman Hospital, Shahid Beheshti University of Medical Sciences, Tehran, Iran; 2 Department of Audiology, Rehabilitation Research Center, School of Rehabilitation Sciences, Iran University of Medical Sciences, Tehran, Iran

**Keywords:** TBS, BMD, T2DM

## Abstract

**Background:**

Despite having higher bone mineral density (BMD) values, type 2 diabetes mellitus (T2DM) patients are at increased risk of fracture. Trabecular bone score (TBS) obtained by evaluating bone microarchitecture might be a more accurate factor for determining bone strength in T2DM patients. In this study, we aimed at investigating the mean values of lumbar spine (LS) TBS, LS-BMD, and femoral neck BMD in T2DM patients and controls, as well as the ability of LS-TBS and BMD in distinguishing between T2DM patients and controls.

**Methods:**

This case-control study was conducted on 150 patients with T2DM (129 women, 21 men) and 484 controls (424 women, 60 men) in Tehran, Iran. LS-TBS along with femoral neck BMD and LS-BMD was computed using dual-energy X-ray absorptiometry images. Diagnostic accuracy and discriminative capacity of LS-TBS, femoral neck BMD, and LS-BMD between the case and control groups were assessed.

**Results:**

T2DM patients showed significantly lower LS-TBS values compared to the control group in the total population and in women. However, in T2DM patients, femoral neck BMD and LS-BMD were found to be significantly higher in the total population and in men, respectively, compared to the control group. Based on area under the curve (AUC) and after adjusting for age and BMI, TBS, LS-BMD, and femoral neck BMD were shown to have the acceptable ability in distinguishing T2DM patients and controls.

**Conclusion:**

Besides higher BMD and lower TBS values in T2DM patients compared to controls, a similar acceptable discriminative ability of LS-TBS, LS-BMD, and femoral neck BMD in differentiating between T2DM patients and controls was observed in the total population and in women.

## Background

It is now well established that patients with type 2 diabetes (T2DM), especially those with poor glycemic control and chronic complications, are at increased risk for developing osteoporosis and subsequent fractures, in particular at the hip [1–3].

Currently, measuring bone mineral density (BMD) by dual X-ray absorptiometry (DXA) is an established method and gold standard for osteoporosis diagnosis and fracture risk prediction [4, 5]. However, despite T2DM patients’ increased susceptibility to fracture compared to non-diabetics, there are many studies demonstrating higher mean BMD values in T2DM patients [6–8], suggesting an underestimation of osteoporosis in T2DM patients [9].

Bone density and bone quality together impact bone strength [10]. Therefore, a reduction of bone quality, which cannot be assessed by BMD, may be the cause for the observed increased risk of fracture in T2DM patients [9, 11]. The bone quality can be assessed through an analysis of iliac bone biopsy, which is the gold standard but also invasive and expensive. Bone microarchitecture (as a component of bone quality) can also be evaluated using the trabecular bone score (TBS), a noninvasive gray-level texture parameter that is widely used and cost-effective and calculated based on DXA images previously obtained for determining BMD [12, 13]. TBS uses experimental variograms of 2D projection images and thereby provides information about 3D bone characteristics [14], such as the trabecular number, the trabecular separation, and the connectivity density. Higher values of TBS are consistently associated with an increase in bone strength and fracture-resistant microarchitecture, and lower values indicate weak, fracture-prone structure [12, 13, 15].

Previous studies found that TBS is a BMD-independent parameter that captures a larger portion of bone microarchitecture deterioration and diabetes-associated fracture risk than does BMD in T2DM patients [6, 8]. These results are consistent with a cross-sectional study conducted on 477 diabetic and control participants that showed TBS could better discriminate patients with diabetes who are concomitantly undergoing glucocorticoid therapy from the control population than BMD [16]. However, a study by Ebrahimpour et al. on 2263 subjects showed that despite increased BMD values in diabetic patients, the quality of bone microarchitecture assessed by TBS did not significantly differ from non-diabetic subjects [17]. Hence, it remains to be evaluated whether TBS as a BMD-independent parameter could be used for the assessment of bone strength in diabetic patients. In order to fulfill this aim, studies evaluating the diagnostic ability of TBS in comparison with BMD in discriminating T2DM patients and non-diabetic individuals are needed, which, as far as we know, are scarce.

In the current study, we aimed at investigating the mean values of lumbar spine (LS) TBS, LS-BMD, and femoral neck BMD in T2DM patients and controls, as well as the ability of LS-TBS and BMD in distinguishing between T2DM patients and controls in both male and female populations in Tehran, Iran.

## Methods

### Study design and participants

This study was designed as a case control and was conducted among adults who were referred to Resalat Hospital Bone Densitometry Center in Tehran, Iran, during the period August 2016 to June 2018. Subjects were classified into two groups based on their T2DM status: T2DM patients and controls. Of a total of 1129 individuals who had attended for the LS and hip BMD scan at the department, 11 had incomplete data, and of the remaining, 150 patients had T2DM (case group). From the remaining 968 individuals, 484 non-diabetic individuals were selected by a simple randomization method (control group).

### Data collection and measurements

Data collection and measurements were assessed equivalently in the case and control groups. The patient’s electronic medical records were reviewed to collect demographic information, including age, sex, weight, height, medical history, values of femoral neck BMD and LS-BMD, as well as LS-TBS. Demographic data regarding height and weight were collected by qualified personnel working at the department. Body mass index (BMI) was calculated as weight (kg) divided by [height (m)]^2^. DXA scans were obtained using a DXA Hologic device (Hologic Delphi A; Hologic, Bedford, MA, USA) for standard measurements of LS at the level of L1-L4 and femoral neck BMD. BMD was expressed in g/cm^2^. TBS evaluation for LS (L1-L4) was performed at the time of the BMD measurement by reanalyzing DXA images using TBS iNsight software (version 2.2; Medimaps, Geneva, Switzerland). Participants were considered to have T2DM based on their medical history shared by the department’s physician. Records without complete data were excluded, and, finally, 634 subjects entered the study, and a database was generated using SPSS version 20.0. Subjects were classified into two groups based on their T2DM status (T2DM patients and controls).

### Statistical analysis

An analysis was run on all subjects as well as in males and females separately. The continuous quantitative data are shown as the mean ± standard deviation (SD). Between-group data were compared using the independent *t*-test for normally distributed variables and Mann–Whitney U test for variables with non-normal distribution. For assessing the diagnostic accuracy and discriminative capacity of LS-TBS, femoral neck BMD, and LS-BMD between the case and control groups, we used receiver operating characteristic (ROC) curves and calculated area under the curve (AUC). Moreover, statistical modeling was performed for adjustment of possible confounders (age and BMI). The closer to 1 the AUC is, the better the model is. The analysis was performed using the SPSS version 20.0 software, and a *p*-value <0.05 was considered as statistically significant.

## Results

Baseline characteristics of the study population based on their group (T2DM vs. controls) are depicted in Table 1. Of the 634 study subjects, 23.66% (n = 150) were diagnosed with T2DM. The total population, including 87.2% women, had a mean age of 59.49 ±12.53 years and a mean BMI of 28.70 ± 4.99 kg/m^2^, which were significantly higher in the T2DM group compared to the control group. Additionally, there were significant differences between the control and T2DM groups regarding the mean of femoral neck BMD, with higher values in diabetics (0.765 ±0.156 vs.0.739 ±0.151, *p*-value =0.013) and LS-TBS, which was higher in the control group (1.287±0.105 vs. 1.327 ± 0.107, *p*-value <0.001). LS-BMD was also higher in the T2DM group, but the difference was not statistically significant (0.962 ±0.164 vs. 0.930 ± 0.182, *p*-value =0.105).

**Table 1:** Baseline characteristics of study subjects

Variables	Total (n = 634)	Control (n = 484)	T2DM (n = 150)	*p*-value
Age	59.49 ±12.53	58.54 ±13.03	62.54±10.24	0.003
Sex (Female)	87.2%	87.6%	86.0%	0.579
BMI (kg/m^2^)	28.70±4.99	28.32±5.07	29.96 ±4.52	<0.001
LS-BMD	0.937 ±0.179	0.930 ±0.182	0.962±0.164	0.105
Femoral neck BMD	0.745±0.152	0.739±0.151	0.765 ±0.156	0.013
LS-TBS	1.318 ±0.108	1.327 ±0.107	1.287±0.105	<0.001

*BMI* body mass index, *TBS* trabecular bone score, *BMD* bone mineral density, *T2DM* type 2 diabetes mellitus, *LS* lumbar spine

A subgroup analysis for female and male subjects and their baseline characteristics are presented in Tables 2 and 3, respectively. In females, significant differences were observed in mean age and BMI with higher values in the T2DM group. LS-BMD and femoral neck BMD were higher in the T2DM group, but the difference was not statistically significant. LS-TBS was significantly higher in the control group (in females) (1.321 ±0.108 vs. 1.276 ±0.104, *p*-value <0.001), similar to that in the overall population (Table 2). In men, significant differences between the control and T2DM groups were only observed in mean values of BMI and LS-BMD with higher values for the T2DM group (1.056 ±0.110 vs. 0.973 ± 0.185, *p*-value =0.021). Although femoral neck BMD was higher and LS-TBS was lower in the T2DM group in men, the difference was not statistically significant (Table 3).

**Table 2:** Baseline characteristics of female study subjects

Variables	Control (n = 424)	T2DM (n = 129)	*p*-value
Age	51.18 ±12.60	62.51 ±10.08	0.002
BMI (kg/m^2^)	28.59±5.05	30.17±4.51	<0.001
LS-BMD	0.923 ±0.181	0.947 ±0.166	0.452
Femoral neck BMD	0.733±0.151	0.756±0.163	0.054
LS-TBS	1.321 ±0.108	1.276 ±0.104	<0.001

*BMI* body mass index, *TBS* trabecular bone score, *BMD* bone mineral density, *T2DM* type 2 diabetes mellitus, *LS* lumbar spine

**Table 3:** Baseline characteristics of male study subjects

Variables	Control (n = 60)	T2DM (n = 21)	*p*-value
Age	61.07 ±15.60	62.72 ±11.47	0.871
BMI (kg/m^2^)	26.38±4.81	28.66±4.47	0.027
LS-BMD	0.973 ±0.185	1.056 ±0.110	0.021
Femoral neck BMD	0.780±0.143	0.823±0.094	0.187
LS-TBS	1.371±0.090	1.356 ±0.080	0.497

*BMI* body mass index, *TBS* trabecular bone score, *BMD* bone mineral density, *T2DM* type 2 diabetes mellitus, *LS* lumbar spine

Table 4 shows the AUCs with 95% confidence intervals (95% CI) of femoral BMD, LS-BMD, and LS-TBS in an unadjusted model (model 1) as well as in an age and BMI adjusted model (model 2), to illustrate their diagnostic capacity of discriminating T2DM patients and control individuals in the total population as well as in men and women separately. In the unadjusted model, TBS and femoral neck BMD showed significant ability in differentiating between T2DM patients and control individuals in the total population (TBS: AUC 0.600, *p* < 0.001; femoral neck BMD: AUC 0.570, *p* = 0.010) and in women (TBS: AUC 0.613, *p* < 0.001; femoral neck BMD: AUC 0.559, *p* = 0.048). Moreover, after adjusting for age and BMI (model 2), TBS, LS-BMD, and femoral neck BMD were shown to have the significant ability in distinguishing T2DM patients and controls in the total population and in women (AUC > 0.600, *p* < 0.001). LS-BMD (AUC 0.639, *p* < 0.038) in men was the only test with significant discriminative ability in differentiating between T2DM patients and control individuals in both unadjusted and adjusted models.

**Table 4:** The area under the curves (AUCs) with 95% confidence intervals (95% CI) of femoral BMD, LS-BMD and LS-TBS in total population, women, and men based on unadjusted (model 1) and age and BMI adjusted model (model 2)

	Model 1			Model 2		
	AUC	95% CI	*p*-value	AUC	95% CI	*p*-value
TOTAL						
Femoral neck BMD	0.570	0.516–0.623	0.010	0.644	0.597–0.691	<0.001
LS-BMD	0.544	0.491–0.599	0.100	0.640	0.592–0.687	<0.001
LS-TBS	0.600	0.549–0.651	<0.001	0.636	0.588–0.684	<0.001
WOMEN						
Femoral neck BMD	0.559	0.501–0.618	0.048	0.648	0.598–0.699	<0.001
LS-BMD	0.523	0.465–0.581	0.443	0.641	0.590–0.692	<0.001
LS-TBS	0.613	0.558–0.667	<0.001	0.642	0.591–0.693	<0.001
MEN						
Femoral neck BMD	0.597	0.471–0.723	0.130	0.613	0.480–0.747	0.097
LS-BMD	0.670	0.548–0.792	0.006	0.639	0.508–0.770	0.038
LS-TBS	0.540	0.400–0.679	0.577	0.613	0.471–0.756	0.117

*AUC* area under the curve, *95% CI* 95% confidence interval, *TBS* trabecular bone score, *BMD* bone mineral density, *T2DM* type 2 diabetes mellitus, *LS* lumbar spine

## Discussion

This case-control study shows higher values of femoral neck BMD and lower values of LS-TBS in the total population and in women, as well as higher LS-BMD in male T2DM patients, compared to controls. Moreover, LS-TBS was found to have a significant discriminating ability similar to the femoral neck and LS-BMD in differentiating between diabetics and control individuals in the total population and in women.

Previous studies reported higher BMD values in T2DM patients in comparison with healthy individuals, which is in accordance with the results of the current study [6–8, 18]. A meta-analysis on 3437 diabetics and 19,139 controls showed that BMD in diabetics was significantly higher at the femoral neck, hip, and LS in both genders [7]. In the present study, femoral neck BMD but not LS-BMD was significantly higher in the total population as well as in women; however, only LS-BMD showed significantly higher values in male T2DM patients. Increased BMD in T2DM patients might be due to the positive correlation between BMD and BMI and the protective effect of obesity against osteoporosis [19]; however, there is still controversy in this regard; in some cohort studies, higher BMD values in T2DM patients persist even after adjustment for BMI [7].

Despite higher BMD values, many studies reported an increased risk of fracture among T2DM patients [2, 3]. Two meta-analyses including 7,832,213 subjects demonstrated a higher risk of hip fractures in diabetic patients in comparison to the general population [1, 2]. Taken together, these findings suggest that diabetes through different mechanisms may have a direct adverse effects on bone strength, which cannot be reflected by BMD alone [11, 13]. A context of low bone turnover, low bone formation, and accumulation of advanced glycation end-products (AGEs) in the bone matrix, inflammation response, oxidative stress, adipokine alterations, Wnt dysregulation, and increased marrow fat and increased bone porosity are reported as plausible causes for bone fragility in T2DM patients [11, 13]. Moreover, low insulin growth factor-1 (IGF-1) levels, a substance responsible for promoting osteoblasts and collagen synthesis, reported in T2DM patients are probably contributing to higher fracture rates [11]. Hence, it remains to be evaluated if and how these factors may interact with bone microarchitecture and bone quality in T2DM patients and whether the effects of these mechanisms on the bone strength could be detected by BMD and TBS.

Recent evidence suggests that TBS determined by using *gray textures of 2D DXA images and evaluating bone trabecular microarchitecture, a component of bone quality, might be a more accurate determining factor of bone strength and fracture risk, independent of BMD, in T2DM patients [6]. There are some studies that reported significantly lower TBS levels, despite higher mean BMD levels, in T2DM patients than non-diabetics [16], a finding in line with the results of the present study and also confirmed in larger studies such as a study on 29,407 postmenopausal women, including 2356 DM patients in Manitoba, Canada [6], and the Ansung study on 1229 men and 1529 postmenopausal women in Korea (325 men and 370 women with T2DM) [8].

However, there is evidence showing higher TBS values among T2DM patients than controls [20] as well as evidence showing no significant difference in this regard, despite significant higher values of BMD among diabetics, for example, a case-control study by Zhukouskaya et al. on 99 diabetic patients and 107 controls showing that TBS values were not significantly different among DM patients and controls; however, it was lower in patients with DM who suffered fractures [21]. Another population-based study among the Iranian population consisting of 2263 participants aged 60 years and above also showed that mean TBS values were not significantly different among diabetics and normoglycemics [17].

Moreover, we found a gender dissimilarity regarding LS-TBS differences between T2DM patients and controls. A significant difference between LS-TBS values of the two groups was observed only among women, similar to a study by Rianon et al., which was conducted on 153 men and women [22]. The observed gender difference may be due to the relatively small number of men in our sample. However, a recent meta-analysis conducted on 35,546 women and 4962 men reported that patients with diabetes and prediabetes had significantly lower TBS than non-diabetics; however, the difference was greater in women than in men [23]. In addition, a large-scale study among DM and non-diabetic men aged 65 years and older showed no significant association between DM and increased prevalence or incidence of vertebral fracture [24]. This suggests that the relation between DM and fracture risk might be more prominent in women than in men.

Despite the growing body of evidence assessing TBS values in diabetic patients, there are limited studies regarding the ability of TBS vs. BMD in differentiating diabetic patients and control individuals. A study by Xue et al. on 477 diabetic and non-diabetic subjects showed that LS-TBS was more effective in discriminating diabetic patients from control subjects than BMD [16]. In the present study, LS-TBS has been shown to have a significant ability in differentiating between T2DM patients and controls in the total population as well as in women based on AUC and ROC analysis, similar to the femoral neck and LS-BMD in this group (adjusted for age and BMI). In contrast to LS-BMD, the ability of LS-TBS in distinguishing between T2DM patients and controls remains significant after adjustment for age and BMI, which might show that TBS is less affected by age and BMI, which are possible confounding factors in assessing bone characteristics [25–27], although more studies are needed to investigate this notion. A possible explanation for the lower ability of LS-BMD in distinguishing T2DM women and controls before adjustment for age and BMI might be the effect of degenerative effects of age on the LS, which had been shown previously to affect osteoporosis diagnosis in elderly women [28]. In men, LS-BMD was the only acceptable method with a relatively good ability in distinguishing between T2DM patients and controls. The comparatively smaller sample size in the male population may be responsible for the observed differences between the two genders. These findings are consistent with studies that considered BMI as a potential confounder in the relationship between T2DM and microarchitectural abnormalities through different mechanisms, including lower bone formation, elevated serum sclerostin, increased adipocyte markers, and abnormal bone marrow fat composition [25–27].

There are some strengths of the present study, including having a population-based design with a relatively large number of study participants and assessment of LS-TBS and femoral neck and LS-BMD at the same time. Moreover, in the present study, the type of diabetes that can affect bone health through different pathophysiology and therapeutic interventions [13] was considered, and all the diabetic participants were suffering from T2DM. Another strength of the study was adjustments for age and BMI, which are important confounding factors in the bone assessment. However, the present study has several noteworthy limitations. First, the small number of men included in the study limited the power of the analysis. Second, we did not have information regarding prevalent fracture, glucocorticoid consumption, and the duration and severity of diabetes of the participants or medication used by them, which have been demonstrated to affect bone strength in such patients [13]. Third, the diagnosis of DM in the study participants was based on their history, and no laboratory assessment was done confirming the DM diagnosis. Fourth, while it is believed that menopausal status is a possible confounder in assessing bone characteristics, due to lack of data, we were unable to include it in our adjusted model. Finally, considering the cross-sectional design of the study, we did not test the association of LS-TBS and BMD values in T2DM patients and non-diabetic subjects with further outcomes such as fracture, and more longitudinal studies are needed in this area.

## Conclusion

The present study showed higher values of femoral neck BMD and lower values of LS-TBS in the total population and in women, as well as higher LS-BMD in men in T2DM patients, compared with controls. Besides, a similar acceptable discriminative ability of LS-TBS, LS-BMD, and femoral neck BMD in differentiating between T2DM patients and controls was observed in the total population and in women. More studies with longitudinal designs, larger sample sizes, and matched pairs are needed to better address this matter in order to have a better understanding of the clinical applicability of TBS in diabetic and non-diabetic patients.

## Data Availability

The original data is part of the electronic medical records of the Resalat Hospital, Tehran, Iran. Therefore, data is not accessible for the public. Any questions about the analyzed data can be addressed to the corresponding author.

## List of abbreviations

*TBS*: Trabecular bone score
*BMD*: Bone mineral density
*T2DM*: Type 2 diabetes mellitus
DM: diabetes mellitus
*LS*: lumbar spine
*BMI*: body mass index
*AUC*: area under the curve
*ROC*: receiver operating characteristic
*DXA*: dual-energy x-ray absorptiometry
*AGE*: advanced glycation end-products
*IGF*: insulin growth factor
